# Fucoidan from Seaweed *Fucus vesiculosus* Inhibits Migration and Invasion of Human Lung Cancer Cell via PI3K-Akt-mTOR Pathways

**DOI:** 10.1371/journal.pone.0050624

**Published:** 2012-11-30

**Authors:** Hyunkyoung Lee, Jong-Shu Kim, Euikyung Kim

**Affiliations:** 1 Department of Pharmacology and Toxicology, College of Veterinary Medicine, Gyeongsang National University, Jinju, South Korea; 2 Research Institute of Life Science, Gyeongsang National University, Jinju, South Korea; Rutgers University, United States of America

## Abstract

**Background:**

Recently there has been an increased interest in the pharmacologically active natural products associated with remedies of various kinds of diseases, including cancer. Fucoidan is a polysaccharide derived from brown seaweeds and has long been used as an ingredient in some dietary supplement products. Although fucoidan has been known to have anti-cancer activity, the anti-metastatic effects and its detailed mechanism of actions have been poorly understood. Therefore, the aims of this study were to demonstrate the anti-metastatic functions of fucoidan and its mechanism of action using A549, a highly metastatic human lung cancer cell line.

**Methods and Principal Findings:**

Fucoidan inhibits the growth of A549 cells at the concentration of 400 µg/ml. Fucoidan treatment of non-toxic dose (0–200 µg/ml) exhibits a concentration-dependent inhibitory effect on the invasion and migration of the cancer cell via decreasing its MMP-2 activity. To know the mechanism of these inhibitory effects, Western blotting was performed. Fucoidan treatment down-regulates extracellular signal-related kinase 1 and 2 (ERK1/2) and phosphoinositide 3-kinase (PI3K)–Akt–mammalian target of rapamycin (PI3K-Akt-mTOR) pathways. Furthermore, fucoidan decreases the cytosolic and nuclear levels of Nuclear Factor-kappa B (p65).

**Conclusions/Significance:**

The present study suggests that fucoidan exhibits anti-metastatic effect on A549 lung cancer cells via the down-regulation of ERK1/2 and Akt-mTOR as well as NF-kB signaling pathways. Hence, fucoidan can be considered as a potential therapeutic reagent against the metastasis of invasive human lung cancer cells.

## Introduction

Metastasis is a leading cause (up to 90%) of cancer-related deaths. The development of cancer metastasis consists of multiple processes, in which cancer cells first detach from the primary tumor, invade surrounding tissues and intravasate into blood and/or lymphatic systems and extravasate from the vasculature and subsequently settle and colonize at the target organs. Matrix metalloproteinases (MMPs) play a key role in tumor metastasis, where MMPs degrade extracellular matrix (ECM) proteins such as collagen, proteoglycan, elastin, laminin, and fibronectin [1], [2]. Among them, MMP-2 (gelatinase A) and MMP-9 (gelatinase B) are expressed abundantly in various malignant cancers and degrade type IV collagen, which is a major component of the basement membrane. Generally, MMP-2 is over-expressed constitutively in highly metastatic cancers, whereas MMP-9 is induced by some stimulating factors such as inflammatory cytokines, epidermal growth factor and phorbol-12-myristate-13-acetate [3]. Therefore, MMP-2 may play a more important role in the cancer cells migration and invasion.

Lung cancer is one of the most common cancers in the world and a leading cause of cancer death, accounting for more than a million deaths yearly worldwide [4]. In particular, it is an extremely aggressive and metastatic tumor probably due to secreting higher levels of MMPs comparing with other cancers. Hence, the inhibition of MMP-2 in cancer cells appears to be an interesting therapeutic target of highly metastatic lung cancer cells. MMPs expression is regulated by transcriptional factors (AP-1 and NF-kB) through upstream pathways including mitogen activated kinases (MAPKs) and PI3K-Akt pathways [5]. MAPKs consist of three kinases, including extracellular signal-related kinase 1 and 2 (ERK1/2), c-JUN N-terminal kinase/stress activated protein kinase (JNK/SAPK), and p38. MAPKs are implicated in a variety of cellular functions such as proliferation, migration and invasion [3]. Activation of PI3K can stimulate its downstream target, Akt, and then regulate cell growth, adhesion, and invasion [6].

There have been numerous reports investigating the cancer chemopreventive or therapeutic agents from natural products. Many species of marine algae have long been used in food diet and also documented as being used in traditional oriental medicine for over 1000 years [7], [8]. They contain various functional components, such as porphyran, gepsin, alginic acid, and oligosaccharide. Fucoidan, whose molecular weight average is about 20,000, is a sulphated polysaccharide extracted from brown marine algae and consists of L-fucose and sulphate ester groups. It has been previously suggested that fucoidan has various biological activities such as antibacterial [9], antioxidant [10], anti-inflammatory [11], anticoagulant [12], and anti-tumor activities [2]. In C57BL/6 mice transplanted with Lewis lung adenocarcinoma cells, fucoidan from *Fucus evanescens* produced moderate antitumor and anti-metastatic effects and potentiated the anti-metastatic, but not antitumor activities of cyclophosphamide [13]. In addition, fucoidan inhibited MMP-2/9 activities and invasiveness of HT-1080 cells and formation of vascular tubules via suppressing expression and secretion of an angiogenesis factor [14]. However, molecular mechanism of its action has been rarely understood yet.

**Figure 1:**
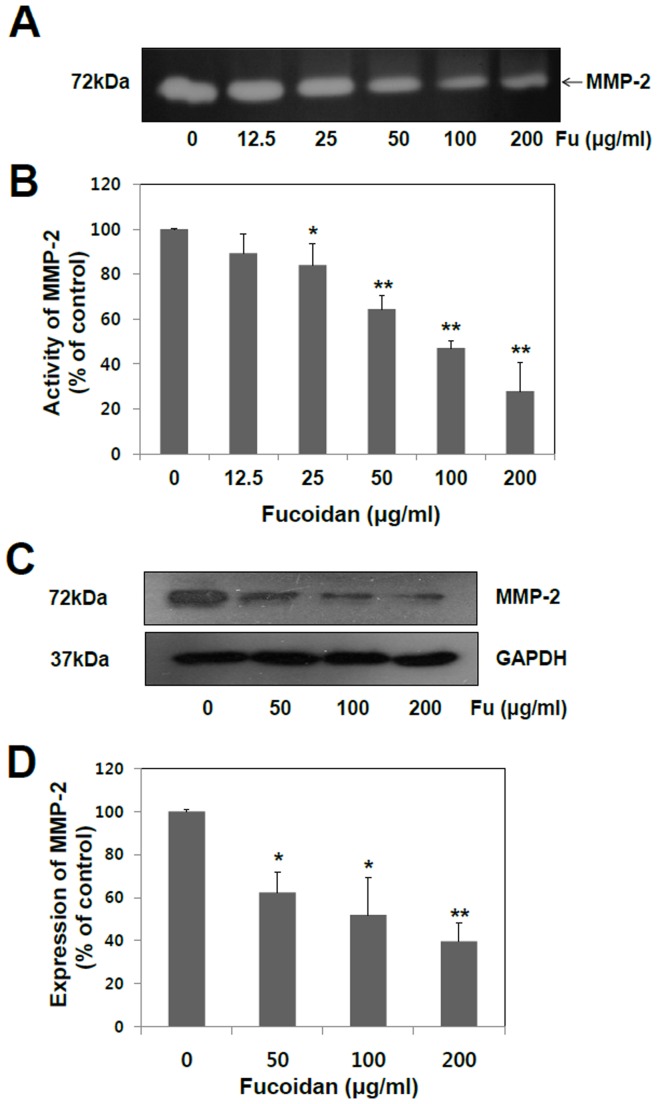
Effect of fucoidan on MMP-2 activity of A549 cells. (A) Subconfluent A549 cells were incubated for 48 hr in the absence or presence of fucoidan (0, 12.5, 25, 50, 100, and 200 µg/ml) in serum-free RPMI media. The conditioned media were collected, and their MMP-2 activity was estimated using gelatin zymography. (B) MMP-2 activity by analysis of zymography was quantified by measuring the band intensities using Image J software. (C) Total cell lysates were subjected to Western blot analysis probed with anti-MMP-2 antibody. (D) Expression of MMP-2 by analysis of western blot was quantified by measuring the band intensities using Image J software. The data shown are the means ± SD of six experiments. Significant difference from control group, *p<0.05 and **p<0.01.

In the present study, it was investigated the effect of fucoidan on migration, invasion and MMP-2 expression of A549 human lung cancer cells and its underlying anti-metastatic mechanisms of action.

## Materials and Methods

### Preparation of Fucoidan and Reagents

Fucoidan extract from seaweed *Fucus vesiculosus* was obtained from Sigma (Sigma, St. Louis, Mo, U.S.A.). Fucoidan powder was dissolved in PBS, then it was sterilized using a 0.45 µm pore filter (Sartorius Biotech GmbH, Gottingen, Germany) and stored as ‘fucoidan extract (20 mg/ml)’ at 4°C until use. MTT reagents and gelatin (G8150) were purchased from Sigma Chemical Co. (St. Louis, MO, USA). RPMI 1640 medium without phenol red, Trypsin–EDTA, penicillin-streptomycin-amphotericin B solution (10,000 U/ml, 10 mg/ml, and 25 µg/ml), fetal bovine serum (FBS), and phosphate buffer solution (PBS) were from Gibco BRL, Life Technologies (USA). LY294002 (PI3K inhibitor), U0126 (ERK1/2 inhibitor), and rapamycin (mTOR inhibitor) were obtained from Tocris Cookson Ltd. (Bristol, UK). For ERK1/2, p38, JNK, Akt, mTOR(Ser2448), and NF-kB, total and/or phosphorylated protein-specific antibodies were purchased from Cell Signaling Technology (Beverly, MA). Invasion assay kit was from BD Biosciences (San Jose, CA, USA).

**Figure 2:**
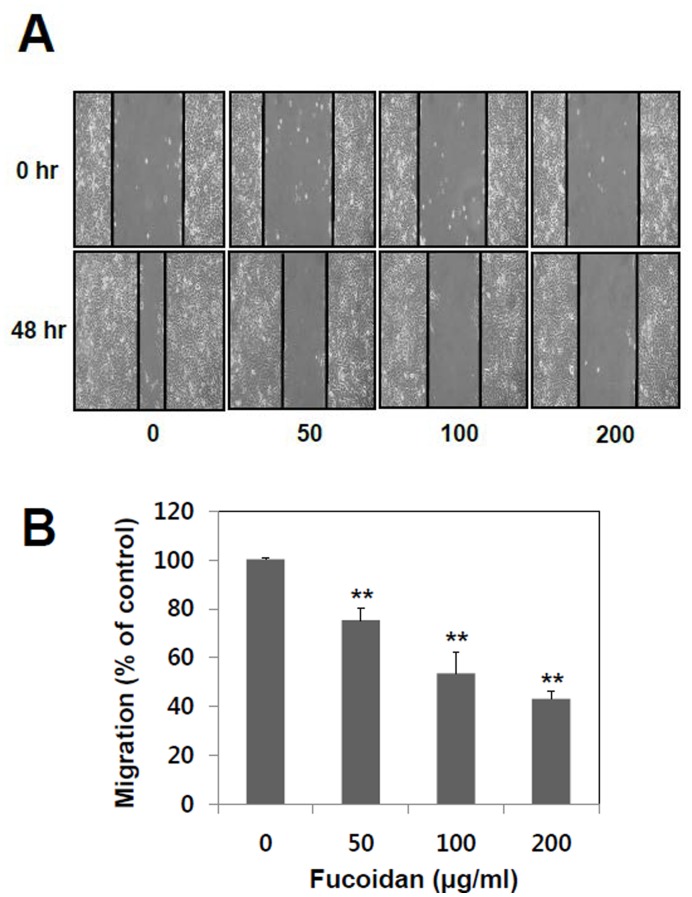
Effects of fucoidan on migration of A549 cells. (A) A549 cells were plated on 12-well and grown to 90% confluence in RPMI containing 10% FBS. Cells were scratched with a pipette tip and then treated with fucoidan (0, 50, 100, and 200 µg/ml). (B) Cell migration distance was estimated by measuring the width of the wound. The data shown are the means ± SD of six experiments. Significant difference from control group, *p<0.05 and **p<0.01.

**Figure 3:**
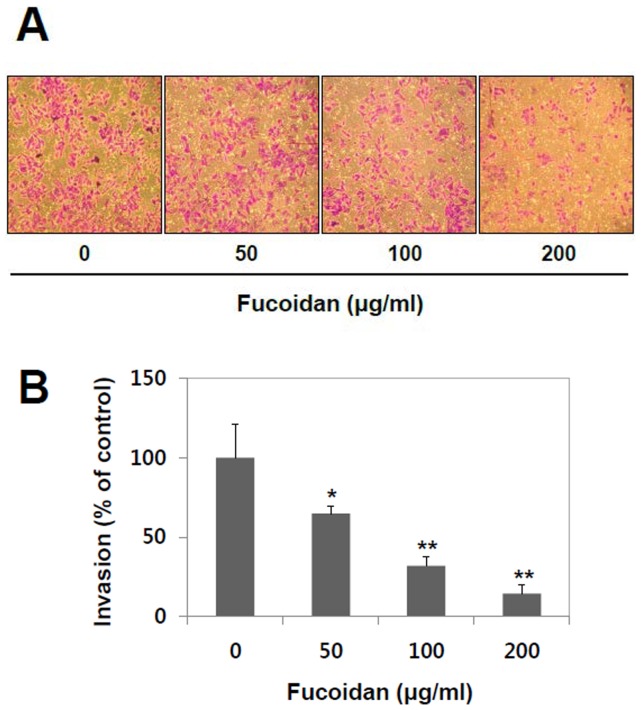
Effect of fucoidan on invasion of A549 cells. (A) Effects of fucoidan on cancer cells invasion were examined using Cell Invasion Assay kit (BD Bioscience, San Jose, CA, USA). For this, A549 cells were seeded onto the upper chamber of Matrigel-coated filter and incubated in the absence or the presence of fucoidan (0, 50, 100, and 200 µg/ml) for 48 hr. The invading cells on the lower surface of the membrane were visualized by crystal-violet staining and observed with light microscope. (B) The amount of invading cells was quantified using Image J software. The data shown are the means ± SD of three experiments. Significant difference from control group, *p<0.05 and **p<0.01.

### Cell Culture

A549 cells from ATCC (American Type Culture Collection, Manassas, VA) were grown in RPMI 1640 supplemented with 10% FBS, 100 µg/ml penicillin-streptomycin-amphotericin B solution at 37 °C in a 5% CO_2_ humidified incubator. Cells were passaged three times a week by treating with trypsin-EDTA and those of after five passages were used for experiments.

**Figure 4:**
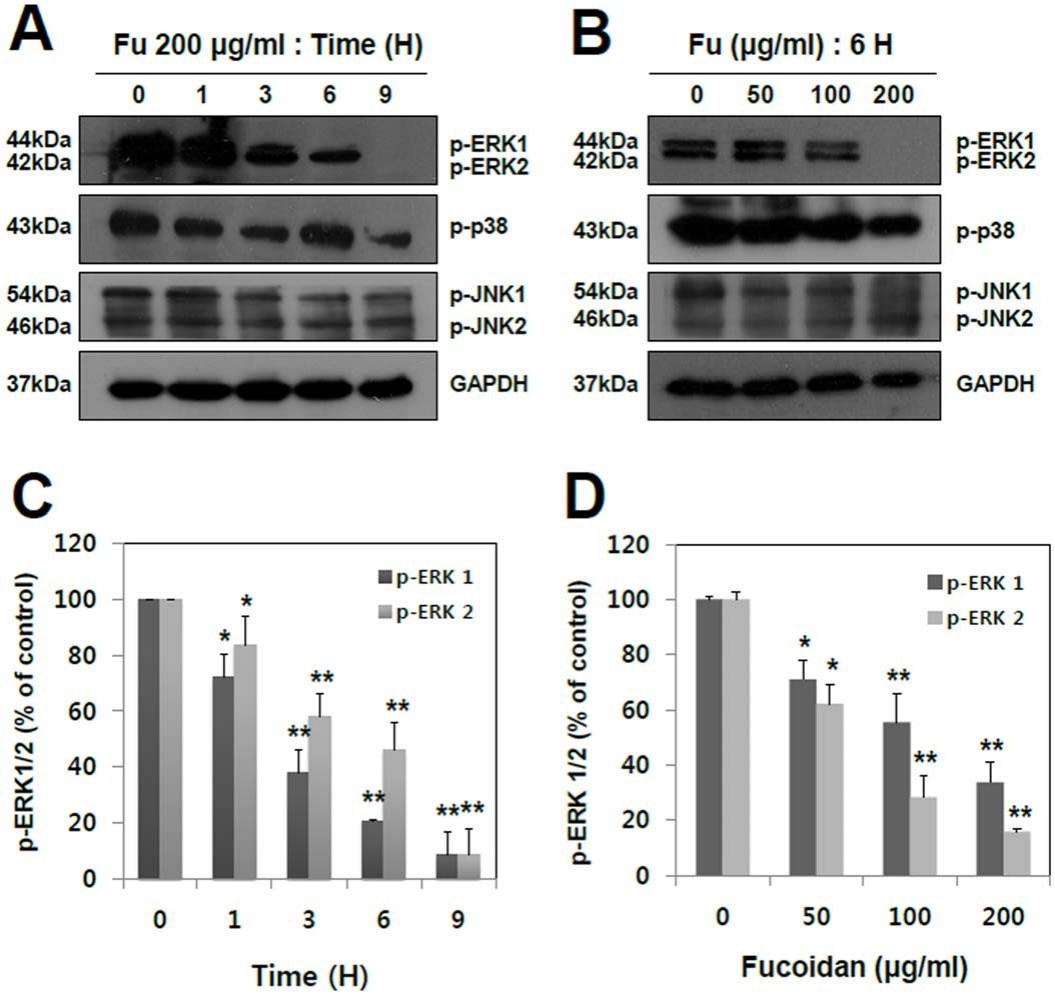
Concentration- and time-dependent effects of fucoidan on phosphorylation of ERK 1/2, p38 and JNK1/2. (A) A549 cells were treated with fucoidan 200 µg/ml for the periods of 0, 1, 3, 6, and 9 hr, respectively. (B) A549 cells were incubated for 6 hr with fucoidan at various concentrations (0, 50, 100 and 200 µg/ml). The levels of phospho-JNK 1/2, phospho-ERK 1/2, and phospho-p38 were analyzed by Western blotting using specific monoclonal antibodies. GAPDH was used as a loading control. (C and D) The phosphorylation level of ERK1/2 was quantified by the analysis with Image J software. The data shown are the means ± SD of three independent experiments. Significant difference from control group, *p<0.05 and **p<0.01.

**Figure 5:**
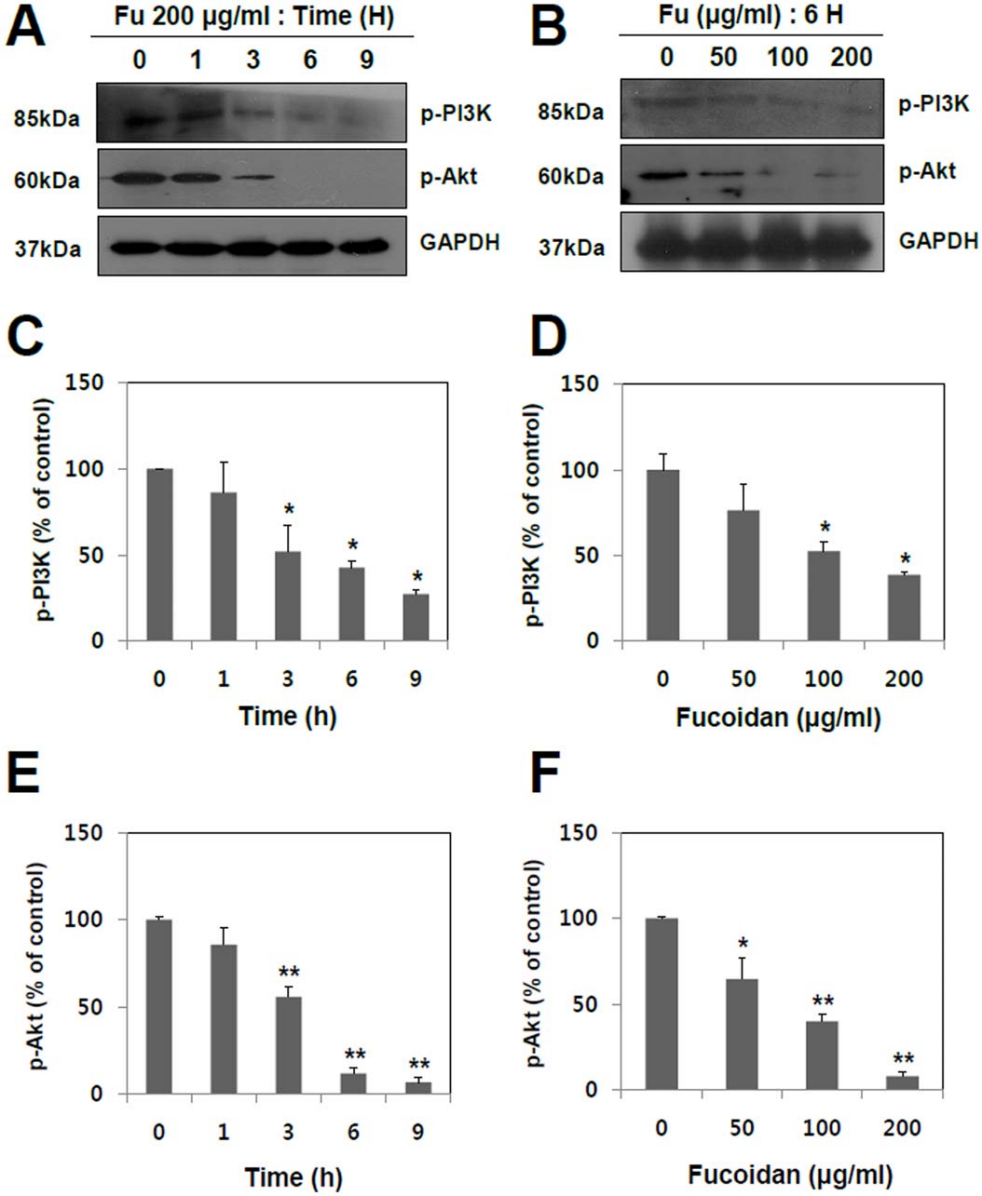
Concentration- and time-dependent effects of fucoidan on PI3K and Akt in A549 human lung cancer cells. (A) A549 cells were treated with fucoidan 200 µg/ml for the periods of 0, 1, 3, 6, and 9 hr, respectively. (B) A549 cells were incubated for 6 hr with fucoidan at various concentrations (0, 50, 100 and 200 µg/ml). The levels of phospho-PI3K and phospho-Akt were analyzed by Western blotting using specific monoclonal antibodies. GAPDH was used as a loading control. The phosphorylation levels of PI3K (C and D) and Akt (E and F) were quantified by the analysis with Image J software. The data shown are the means ± SD of three independent experiments. Significant difference from control group, *p<0.05 and **p<0.01.

**Figure 6:**
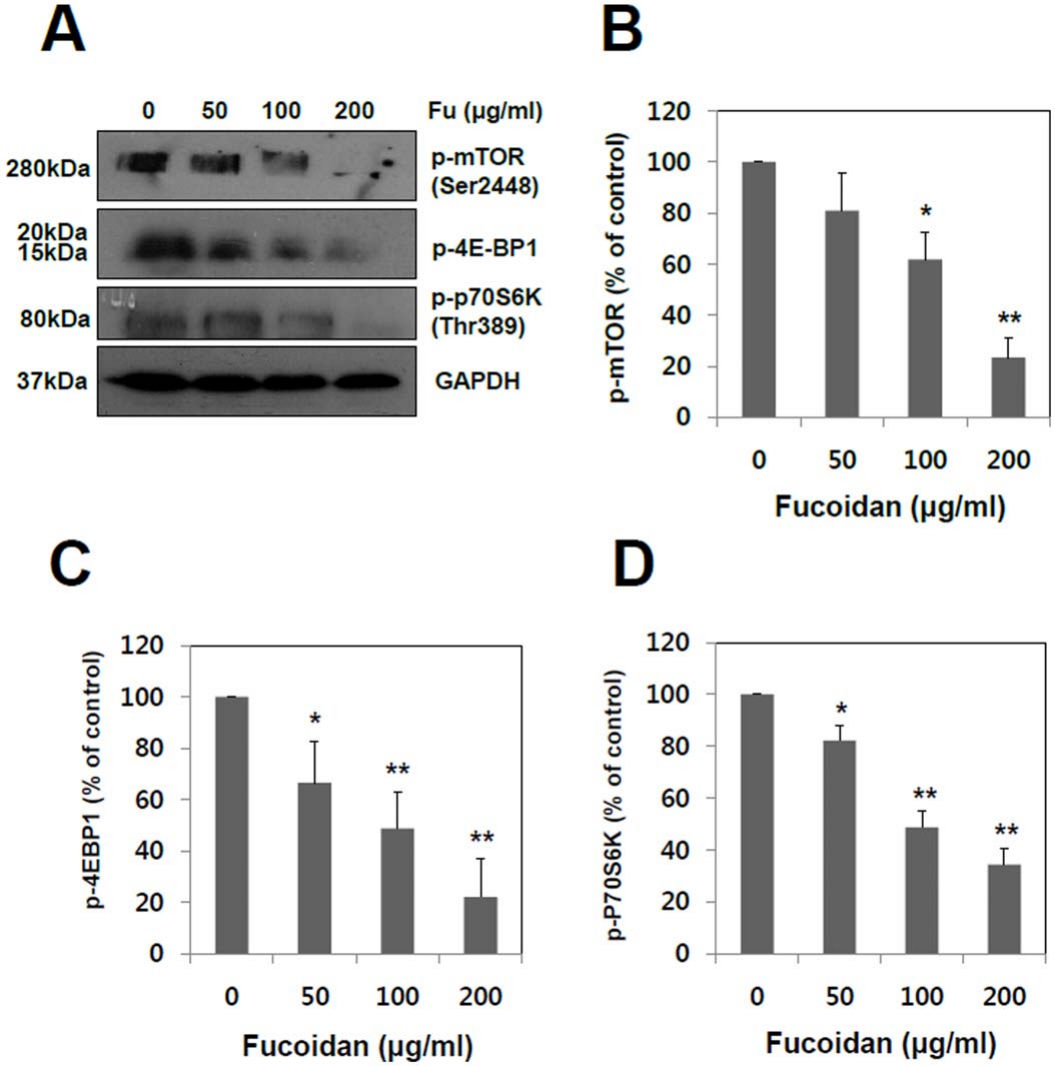
Effect of fucoidan on the phosphorylation of mTOR (Ser2448), p70S6K (Thr389) and 4EBP1 in A549 cells. (A) A549 cells were incubated for 24 hr with fucoidan at various concentrations (0, 50, 100 and 200 µg/ml). The phosphorylation levels of mTOR, p70S6K and 4EBP1 were quantified by the analysis with Image J software. GAPDH was used as a loading control. (B, C and D) The band intensities (phosphorylation levels) of each signaling molecules were quantified by the analysis with Image J software. The data shown are the means ± SD of three experiments. Significant difference from control group, *p<0.05 and **p<0.01.

**Figure 7:**
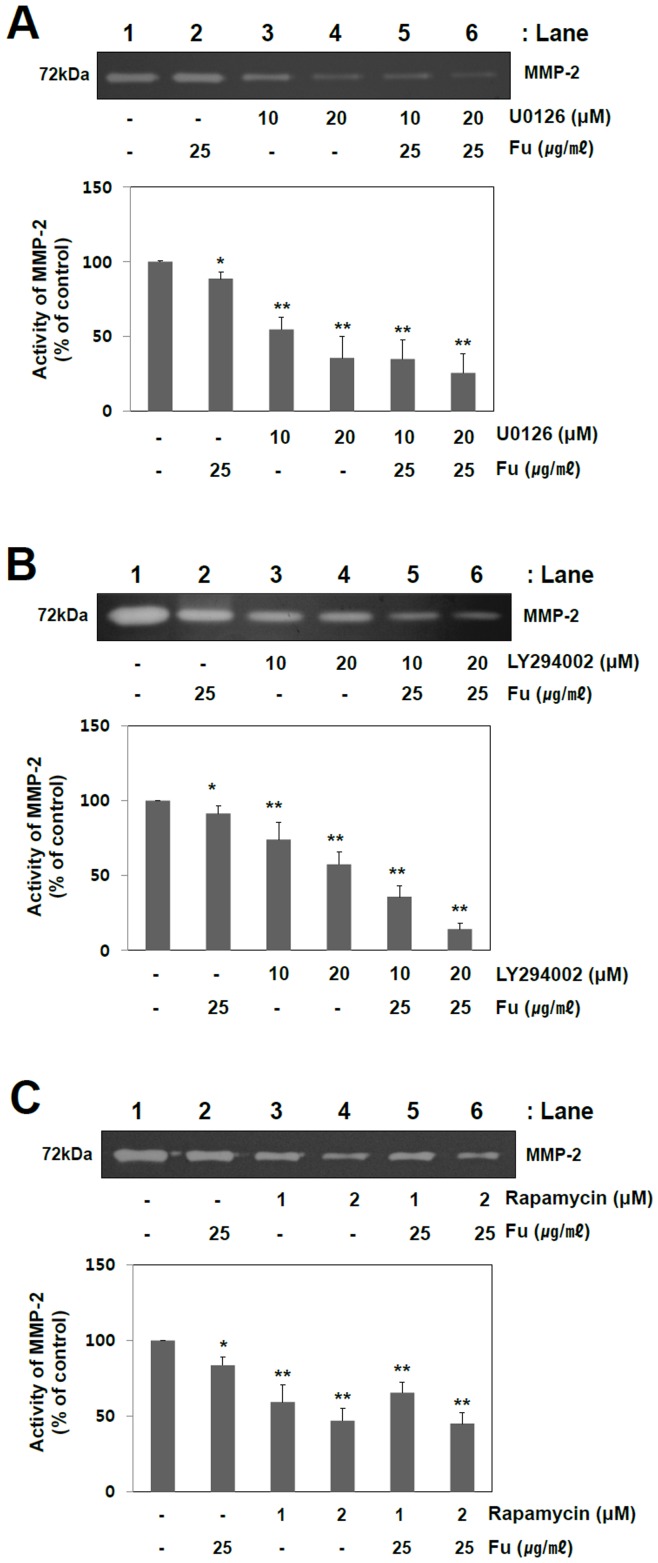
Effect of ERK inhibitor (U0126), PI3K inhibitor (LY294002) and mTOR inhibitor (Rapamycin) in conjunction with fucoidan on MMP-2 activity of A549 cells. (A) Cells were pretreated with U0126 (10 or 20 µM) (A), LY294002 (10 or 20 µM) (B), or rapamycin (1 or 2 µM) (C) for 2 hr and then incubated in the absence or presence of fucoidan (25 µg/ml) for 48 hr. The alterations of MMP-2 activities were quantified by measuring the band intensities using Image J software. The data shown are the means ± SD of three independent experiments. Significant difference from control group, *p<0.05 and **p<0.01.

**Figure 8:**
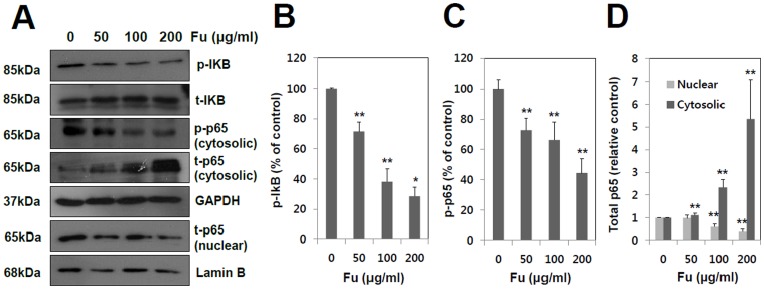
Effect of fucoidan on NF-kB activation in A549 cells. A549 cells were incubated for 24 hr with fucoidan at various concentrations (0, 50, 100 and 200 µg/ml). (A) The cytosolic extract were prepared and analyzed by Western blotting with phospho-IkB and phospho- and total- NF-kB (p65). (B, C and D) The level of phospho-IkB and phosphor- and total NF-kB were quantified by the analysis with Image J software. GAPDH was used as a loading control in cytosolic fraction and Lamin B was loading control in nuclear fraction. The band intensities of each signaling molecules were quantified by the analysis with Image J software. The data shown are the means ± SD of three experiments. Significant difference from control group, *p<0.05 and **p<0.01.

### MTT Assay for Cell Viability

Cell viability was measured by MTT (3-(4,5-dimethyl-2-yl)-2,5-diphenyltetrazolium bromide) reduction assay as described previously [15]. Briefly, A549 cells were incubated at a density of 4×10^4^ cells/well in 24-well plates for 24 h. The cells were treated with various concentrations of fucoidan for 48 h. Then, MTT dye (5 mg/ml) was added to the cells and they were incubated for additional 3 h. After the medium was removed, DMSO was added to the cells for the solubilization of generated formazan salts. The amount of formazan salt was determined by measuring the optical density (OD) at 540 nm using GENios® microplate spectrophotometer (PowerWave^TM^XS, BioTek Instruments, Inc., Winooski, USA). Relative cell viability of treatment was calculated as a percentage of vehicle-treated control ([OD of treated cells – OD of blank/OD of control – OD of blank] × 100).

### Wound Migration Assay

Cell migration assay was performed using 12-well plates as previously described [16]. A549 cells were seeded into 12-well plates (1×10^5^ cells/ml) and grown to 80∼90% confluence for the experiment. After aspirating the medium, cells were scraped with a 200 µl sterile pipette tip to create a straight scratch. They were washed twice with PBS to remove cellular debris and then replaced with complete RPMI 1640. A549 cells were treated with fucoidan (0, 50, 100, and 200 µg/ml) and incubated for 48 hr. Cell migration into the wound area was photographed at the stages of 0 hr and 48 hr, respectively, for the image analysis of each treatment. The level of cell migration was determined using a Hewlett-Packard scanner and NIH Image software (Image J), and then it was expressed as a percentage of each control for the mean of wound closure area.

### Cell Invasion Assay

The invasive behavior of A549 cells was tested using cell invasion chamber kit (BD Bioscience, San Jose, CA, USA) [17]. A549 cells were resuspended in a serum-free RPMI 1640 medium (5×10^4^ cells/200 µl) in the absence or the presence of fucoidan (0, 50, 100 and 200 µg/ml). The cells were seeded onto the upper chamber of Matrigel-coated filter and a RPMI 1640 containing 10% FBS was added 500 µl in the lower chamber. After 48 hr incubation, the non-invading cells were removed from the upper surface of the filter membrane. The invading cells on the lower surface of the filter membrane were stained with crystal violet for 1 hr and rinsed with water and dried. The amount of invading cells on the lower surface of filter membrane was determined using a light microscope and NIH Image software (Image J).

### Gelatin Zymography

MMP-2 activity was determined by gelatin zymography [18]. Briefly, A549 cells were seeded (1×10^5^ cells/well) and allowed to grow to confluence for 24 hr and maintained in RPMI 1640 with 10% FBS. The cells were washed with PBS and incubated with various concentrations of fucoidan (0, 50, 100, and 200 µg/ml) in serum-free RPMI 1640 for 48 hr. The supernatant was collected and mixed with non-reducing sample buffer, then electrophoresed in 10% polyacrylamide gel containing 0.1% (w/v) gelatin. After the electrophoresis, gel was washed for 30 min twice with 2.5% Triton X-100 and incubated for additional 18 hr at 37°C for the enzymatic reaction of MMPs in zymography reaction buffer (200 mM NaCl, 10 mM CaCl_2,_ 50 mM Tris-HCl, pH 7.4). The gel was then stained with Coomassie blue R-250 (0.125% Coomassie blue R-250, 50% methanol, 10% acetic acid) and destained (methanol/acetic acid/water, 40/10/50, v/v/v).

### Preparation of Cell Lysates

After fucoidan treatments, A549 cells were rinsed twice with ice-cold PBS, and then added with 100 µL of lysis buffer (50 mM Tris-HCl, pH 7.4, 1% NP-40, 0.25% sodium deoxycholate, 150 mM EDTA, 1 mM PMSF, 1 mM sodium orthovanadate, 1 mM NaF, 1 mM Na_3_VO_4_, 1 µg/ml aprotinin, 1 µg/ml leupeptin, 1 µg/ml pepstatin). The lysis reaction was performed on ice with rocking for 3 min and the cells were scraped using rubber policeman into Eppendorf microcentrifuge tubes. The scraped cells were allowed to lyse for additional 30 min on ice with periodic vortexing. Cell debris was removed by centrifugation (22,000×*g*, at 4°C for 30 min) and the resulting supernatant was collected and determined for its protein concentration using a Bio-Rad protein assay reagent (Bio-Rad, C.A. USA). Samples were cooked with SDS sample buffer in boiling water for 5 min.

### Western Blotting

The protein components (50 µg) were separated on 12% SDS-polyacrylamide gel, transferred to polyvinylidene difluoride membranes (Bio-Rad, C.A. USA), and subsequently subjected to immunoblot analysis using specific primary antibodies for overnight at 4°C. After washing, the membranes were incubated with horseradish peroxidase-conjugated secondary antibody (Cell Signaling Technology, Beverly, MA) for 1 hr at room temperature. The blots were then visualized by using the enhanced chemiluminescence method (ECL, Amersham Biosciences, Buckinghamshire, UK) on blue light-sensitive film (Fujifilm Corporation, Tokyo, Japan). If necessary, the blotted membranes were stripped and reprobed using other primary antibodies. Densitometry analysis was performed with a Hewlett-Packard scanner and NIH Image software (Image J).

### Statistical Analysis

The results are expressed as the mean ± standard deviation (S.D.). One-way analysis of variance (ANOVA) was used to evaluate the significance of difference between the two mean values. *P<0.05 and **P<0.01 were considered to be statistically significant.

## Results

### Fucoidan Inhibits the Activity of MMP-2 in A549 Human Lung Cancer Cells

In order to determine non-cytotoxic concentration of fucoidan, A549 cells were incubated with various concentrations of fucoidan (0–1000 µg/ml) for 24 hr or 48 hr, and then cell viability was determined using MTT assay (data not shown). Fucoidan did not show any significant toxic effect on A549 cells within the concentration range of 0–200 µg/ml. At higher concentrations (400–1000 µg/ml), however, fucoidan inhibited cell proliferation and decreased cell viability in a concentration-dependent manner. Therefore, the present study applied the treatment of fucoidan at concentrations equal to or lower than 200 µg/ml, at which no significant cytotoxic effect of A549 cells was observed.

The degradation of extracellular matrix (ECM) is crucial for cellular invasion, indicating the inevitable involvement of matrix-degrading proteinases for the process. Hence, it was assessed the effect of fucoidan on MMP-2 activity, as a key molecule of ECM degradation, by gelatin zymography. As shown in Figure 1A, fucoidan suppressed MMP-2 activity in a concentration-dependent manner. On the other hand, the impact of fucoidan on the MMP-9 activity was inconclusive, since there is only an extremely low level of intrinsic MMP-9 activity detectable in A549 cells (data not shown). MMP-2 activity was reduced by 36%, 53%, and 72% at 50, 100, and 200 µg/ml, respectively, after treatment with the fucoidan for 48 hr (Fig. 1B). The results obtained by zymography were further confirmed by Western blot analysis. MMP-2 expression was gradually decreased with increasing concentrations of fucoidan (Fig. 1C). The protein level was reduced by 38%, 48%, and 60% at 50, 100, and 200 µg/ml, respectively. The reduction of MMP-2 activity was probably due to the decrease in MMP-2 expression (Fig. 1B and 1D).

### Fucoidan Suppresses the Migration of A549 Cells

Cell migration is a measure of metastatic potential of cancer cells. Effects of fucoidan on cell migration were investigated using a wound healing assay. A549 cells treated with fucoidan showed reduction of migration (Fig. 2A). As shown in Figure 2B, cell migration was inhibited by up to 25%, 46%, and 57% at 48 hr in the presence of 50, 100, and 200 µg/ml of fucoidan, respectively. Collectively, these data demonstrated that fucoidan may suppress the migratory property of lung cancer cells.

### Fucoidan Inhibits the Invasion of A549 Cells

To elucidate inhibitory effect of fucoidan on invasion of A549 cells, it was tested using invasion chamber kit (BD Bioscience, San Jose, CA, USA), in which cells invade across extracellular matrix, such as Matrigel-coated filter in chamber. As illustrated in Figure 3A, the number of cells with invasion (crystal-violet staining) reduced remarkably in a concentration-dependent manner, suggesting fucoidan can inhibit the invasion of A549 cells. The inhibitory effects of fucoidan on cancer cell invasion were 35%, 68% and 86% at the concentrations of 50, 100, and 200 µg/ml of fucoidan, respectively (Fig. 3B). These results indicated that fucoidan can directly inhibit the invasive potential of lung cancer cell.

### Fucoidan Inhibits the MMP-2 Activity by Blocking ERK1/2 and PI3K-Akt Pathways in A549 Cells

Our findings showed that fucoidan dramatically inhibits migration, invasion and MMP-2 activity in A549 cells. However, the signal mechanisms responsible for the inhibitory effect of fucoidan on metastasis remain to be elucidated. Several evidences suggest that MAPKs (JNK 1/2, ERK 1/2, and p38) play important roles in cancer cell migration, invasion, and MMP-2 activity [5]. Considering the strong anti-metastatic potential of fucoidan, it was tested whether it can regulate the signal transductions of MAPKs in metastatic lung cancer cells. The results of Figure 4 showed that fucoidan reduced phosphorylation of ERK1/2 in a concentration- and time-dependent manner, whereas it had almost no or marginal, if any, effect on p38 and JNK1/2. As shown in Figure 4A and 4C, a time-dependent study demonstrated that fucoidan progressively reduced phosphorylation of ERK1/2 from 1 hr to 9 hr after the treatment. Figure 4B and 4D showed that fucoidan significantly inhibited phospho-ERK1/2 in a concentration-dependent manner with a maximum inhibitory effect at the highest concentration (200 µg/ml). In addition to MAPKs, the cell signaling of PI3K/Akt has been also proposed as a key player in the regulation of MMP-2 activity [19]. Our current study revealed that fucoidan markedly inhibited the phosphorylations of PI3K and its downstream target, Akt, in a concentration- and time-dependent manner (Fig. 5). Based on these results, fucoidan potently inhibited the metastatic activity of human lung cancer cells, possibly by the suppressions of ERK1/2 and PI3K-Akt pathways.

### Fucoidan Suppresses mTOR Signaling

It has been reported that PI3K is associated with a number of cellular processes including proliferation, differentiation, apoptosis, cell motility, invasion and angiogenesis [20], [21]. The signaling of mTOR has been emerged especially as a critical effecter of PI3K signal transduction pathway in various human cancers [22]. Based on our finding that fucoidan suppresses PI3K-Akt pathway in human lung cancer cells, it was examined whether fucoidan modulates mTOR and its downstream signaling molecules. As shown in Figure 6A and 6B, fucoidan inhibited the phosphorylation of mTOR in a concentration-dependent manner. Additionally, 4E-BP1 and p70S6K, two immediate downstream targets of mTOR and indicators of mTOR activity, were also significantly suppressed (Fig. 6C and 6D).

### Fucoidan Inhibits MMP-2 Activity by Blocking ERK1/2 and PI3K-Akt-mTOR Pathways in A549 Cells

To confirm whether the inhibition of MMP-2 activity by fucoidan mainly rely on its suppression of ERK1/2 and PI3K-Akt-mTOR pathways, A549 cells were pretreated in the presence or absence of U0126 (an ERK inhibitor), LY294002 (a PI3K inhibitor) or rapamycin (a mTOR inhibitor) for 2 hr, then examined for the effects of fucoidan treatment (Fig. 7). Compared with the respective vehicle controls, individual treatments with U0126 (Fig. 7A, lane 3 and 4), LY294002 (Fig. 7B, lane 3 and 4) or rapamycin (Fig. 7C, lane 3 and 4) gave a significant reduction of MMP-2 activity. The results are consistent with those of fucoidan experiments of the present study in that MMP-2 inhibition can be mediated by the modulations of ERK1/2, PI3K-Akt and/or mTOR pathways. Describing in detail, the cotreatment of U0126 (10 µM and 20 µM) and fucoidan further reduced the MMP-2 activity by 66% and 75% compared with U0126 alone (46% and 65%) (Fig. 7A). In the same manner, the combined treatments of LY294002 and fucoidan additionally suppressed the MMP-2 activity by 65% and 86% in comparison with LY294002 alone (27% and 43%) (Fig. 7B). However, unlike others, rapamycin and fucoidan cotreatment did not give an additional decrease of MMP-2 activity compared with rapamycin alone (Fig. 7C). It is not yet clearly understood why there was neither additive nor synergistic effects of rapamycin with fucoidan. In summary, the inhibition of MMP2 by fucoidan treatment is possibly exhibited by the modulations of ERK1/2 and/or PI3K-Akt-mTOR pathways. These results proposed the therapeutic potential of fucoidan as an anti-metastatic agent in human lung cancer patients.

### Fucoidan Reduces the Nuclear Translocation of NF-κB

NF-κB pathway has been demonstrated to rely upon sequentially activated kinase cascades [23]. On activation, NF-κB translocates from cytoplasm into nucleus and regulates the expression of a wide variety of target genes, including MMPs [5]. Since NF-κB can play a key role in MMP-2 gene expression, the effects of fucoidan was assessed on IκBα and NF-κB signal transduction. As shown in Figure 8A and 8B, fucoidan inhibited the phosphorylation of IκBα in a concentration-dependent manner, whereas the total IκBα was inversely increased by fucoidan. Being consistent with this, phospho-p65, an indicator of NF-κB activation through IκBα degradation, was decreased by the treatment of fucoidan (Fig. 8A and 8C), which is accompanied by the increase of total p65 in the cytosolic fraction and its decrease in nuclear fraction (Fig. 8A and 8D). The results demonstrated that fucoidan significantly inhibited activation of NF-κB in A549 cells.

## Discussion

It is well known that MMPs provide an access for tumor cells to the vascular and lymphatic vessels, which support tumor growth and constitute an escape route for further dissemination [24]. Furthermore, MMPs promote malignant transformation in early stages of cancer and suppress cancer cell apoptosis and enhance angiogenesis as well as destroy chemokines balance by host anti-tumor defense system [25]. Accordingly, blocking of MMPs activity and expression may lead to the development of effective therapy in metastatic cancer patients. Although many scientists have made great efforts to develop potential anticancer agents of MMPs inhibitors, most clinical trials have not been successfully completed yet [26].

In search of efficacious metastatic suppressors, a wide variety of natural products and their pharmacological ingredients can be a good source of finding promising candidates, having several advantages over synthetic chemicals on side effects and pharmacophore diversity. Some of them from various natural resources have been already characterized as potential therapeutics with anti-metastatic activity [27]. Among them, fucoidan has been revealed to possess anti-proliferative and cytotoxic effects on MCF-7 breast cancer cells, but not human mammary epithelial cells (HMECs) [28]. Recently, fucoidan has been demonstrated to inhibit metastasis via MMP-2/−9 suppression as well as subdue the expression and secretion of a vascular endothelial growth factor (VEGF) [14]. The anti-metastatic activity of fucoidan was also proven in the animal model of experimental transplanted Lewis lung carcinoma (LLC) [13]. However, the inhibitory mechanism on cancer metastasis has not been clearly elucidated in detail yet. For the first time, in this study, it is at least partly identified the anti-metastatic molecular mechanism of fucoidan.

The status of cell viability was assessed using MTT assay in the absence or presence of various concentrations of fucoidan. Fucoidan inhibited cell growth at 400 µg/ml but not at between 0 to 200 µg/ml, at which it effectively inhibited migration and invasion without significant cytotoxic effect. Therefore, the anti-invasive and anti-migratory effects of fucoidan observed from the present study were not dependent on its anti-proliferation effect. The enhancement of MMP-2 activity has been well characterized in highly metastatic human lung cancer cells [29]. However, MMP-9 is still inconclusive, since there is only an extremely low level of MMP-9 detected in A549 human lung cancer cells. Based on our present study, the anti-metastatic effects of fucoidan appear to be mediated by its inhibitory activity on MMP-2. (Figs. 1, 2 and 3). Our results show that fucoidan effect is more like the suppression of MMP-2 secretion and/or expression rather than the direct inhibition of its activity.

ERK1/2 pathway is involved in the invasive or migratory behavior of a number of malignancies, such as colon cancer, melanoma, breast cancer, and prostate cancer and this is well-established in previous studies [30]–[32]. Moreover, ERK1/2 regulates focal adhesion and cytoskeletal reorganization via the phosphorylations of specific cytoskeletal and focal adhesion proteins, including paxillin, FAK, myosin light chain kinase [33]. As shown in Figure 4, the anti-metastatic effect of fucoidan is owing to its inactivation of ERK1/2 pathway in A549 human lung cancer cells. In addition to ERK1/2, the PI3K-Akt signal pathway has been shown to regulate the invasion and metastasis of non-small-cell lung cancer (NSCLC) as well as the development and progress of various other tumors. That is to say, PI3K over-expression is highly correlated with the development, invasion, and metastasis of NSCLC [34]. Several other studies have also shown that targeting of PI3K-Akt signaling pathway with antisense, siRNA or small molecule inhibitors results in the down-regulation of tumor invasion and tumorigenesis in malignant cancer cells [6], [35]. The results of Figure 5 exhibited that fucoidan inhibits the phosphorylation of PI3K-Akt in time- and concentration-dependent manners.

Mammalian target of Rapamycin (mTOR) is a serine/threonine protein kinase that regulates cell growth and proliferation, cell motility, cell survival, protein synthesis, and transcription [36], [37]. The dysregulation of mTOR pathway can be observed in human diseases, especially certain cancers, and there are also some mTOR inhibitors, which are beginning to be used in the treatment of cancer [22]. PI3K and Akt are well known upstream regulators of mTOR signaling pathway in mammalian cells. As an evidence of this notion, siRNA-mediated gene silencing of PI3K and Akt inhibited the activation of p70S6K1 (a downstream target of mTOR), and subsequently led to the suppression of migration, invasion and proliferation [38]. It has also been reported that mTOR pathway regulates MMP-2 and -9 expressions and angiogenesis through modulating VEGF gene expression. The treatment of rapamycin (a mTOR inhibitor) and siRNA-mediated mTOR knockdown inhibit tumor cell invasion as well as the secretions of MMP-2 and uPA [21], [39]. As shown in Figure 6, fucoidan treatment significantly decreased the phosphorylations of mTOR and its downstream target molecules, such as p70S6K1 and 4EBP1. The present study demonstrated that fucoidan effectively down regulates the expression of MMP-2 through the inhibitions of PI3K-Akt-mTOR as well as ERK1/2 signaling pathways in A549 human lung cancer cells. To further support these findings, MMP-2 activity was examined upon the cotreatments of fucoidan with ERK1/2, PI3K or mTOR inhibitor. The MMP-2 suppression induced by the cotreatment is more likely synergistic than additive effect, except mTOR inhibitor. (Fig. 7). Interestingly, unlike other ones, rapamycin co-treatment with fucoidan showed neither synergistic nor additive effect, which is not yet clearly understood. One of the possible interpretations might be the intrinsic limit of efficacy of mTOR signaling pathway in the regulation of MMP2 activity.

NF-kB and AP-1 are transcription factors that regulate the expressions of numerous genes associated with many important biological and pathological processes, including cancer. It has been reported that the Inhibition of NF-kB and AP-1 results in the suppression of tumor initiation, promotion and metastasis [33], [40]. MMP gene expressions are also regulated by NF-kB and AP-1, hence the blocking of these transcription factors is likely to be a promising strategy for cancer therapy aiming at the down regulation of MMP genes [41]. As shown in Figure 8, fucoidan treatment prohibited the degradation of I_k_Bα protein and the translocation of NF-kB p65 to the nucleus.

In conclusion, it is the first time that fucoidan is revealed to inhibit MMP-2 activity via the suppression of PI3K-Akt-mTOR and NF-kB signaling pathway in A549 lung cancer cells, resulting in the down regulation of cancer cell migration and invasion. Further *in vivo* study and clinical investigations are necessary for the application of fucoidan as a novel therapeutic agent and alternative remedy in metastatic cancer patients. By any measure, fucoidan looks promising as an efficacious anti-metastatic reagent with its low side effect in normal cells.
